# Rif1 and Exo1 regulate the genomic instability following telomere losses

**DOI:** 10.1111/acel.12466

**Published:** 2016-03-22

**Authors:** Yuan Xue, Marcus E. Marvin, Iglika G. Ivanova, David Lydall, Edward J. Louis, Laura Maringele

**Affiliations:** ^1^ Newcastle University, Institute for Cell and Molecular Biosciences Institute for Cell and Molecular Biosciences (ICaMB)Newcastle upon TyneUK; ^2^ Department of Genetics, Centre for Genetic Architecture of Complex TraitsUniversity of LeicesterLeicesterUK

**Keywords:** checkpoints, chromosome alterations, Exo1, PAL, Rif1, senescence, telomeres

## Abstract

Telomere attrition is linked to cancer, diabetes, cardiovascular disease and aging. This is because telomere losses trigger further genomic modifications, culminating with loss of cell function and malignant transformation. However, factors regulating the transition from cells with short telomeres, to cells with profoundly altered genomes, are little understood. Here, we use budding yeast engineered to lack telomerase and other forms of telomere maintenance, to screen for such factors. We show that initially, different DNA damage checkpoint proteins act together with Exo1 and Mre11 nucleases, to inhibit proliferation of cells undergoing telomere attrition. However, this situation changes when survivors lacking telomeres emerge. Intriguingly, checkpoint pathways become tolerant to loss of telomeres in survivors, yet still alert to new DNA damage. We show that Rif1 is responsible for the checkpoint tolerance and proliferation of these survivors, and that is also important for proliferation of cells with a broken chromosome. In contrast, Exo1 drives extensive genomic modifications in survivors. Thus, the conserved proteins Rif1 and Exo1 are critical for survival and evolution of cells with lost telomeres.

## Introduction

Human somatic cells have insufficient telomerase activity to repair telomeres, which shorten during DNA replication and other events. Checkpoint pathways detect short and damaged telomeres, leading to a permanent cell cycle arrest, called replicative senescence (Shay & Wright, 2000). Whereas the accumulation of senescent cells in tissues most likely plays a driving role in the aging process (van Deursen, 2014), escape from senescence may lead to cancer (Zou *et al*., 2009). Consistent with this hypothesis, replicative senescence is induced by p53 and other checkpoint proteins inactivated in cancers (Shay *et al*., 1991; Sugrue *et al*., 1997; Schmitt *et al*., 2002). Moreover, cancers may originate from cells with telomere attrition (Meeker *et al*., 2004) or sometimes with acute telomere losses, due to chromosome fragmentation (Štafa *et al*., 2014).

The classical view is that both checkpoint inactivation and telomerase reactivation are required to successfully bypass replicative senescence (Shay *et al*., 1991; Wright & Shay, 1995). However, some cancer cells retain relevant checkpoint activity, for example those able to senesce when treated with telomerase inhibitors (Saretzki, 2003). Moreover, telomerase and/or long telomeres are not essential for proliferation of cancer cells, since telomerase (and checkpoint)‐knockout mice can develop malignant tumours and metastasis (Artandi *et al*., 2000; Bojovic & Crowe, 2013). Very short telomeres are also found in human cancer (Xu & Blackburn, 2007). These data show that cells can undergo malignant transformation and metastasis, despite short telomeres. To do so, they should be able to tolerate telomere losses and the associated genomic instability, and also to escape the DNA damage checkpoint control, through yet unknown mechanisms. One of the best models to identify such mechanisms is the PAL system, consisting of budding yeast cells unable to maintain telomeres, similarly to most human somatic cells (Maringele & Lydall, 2004b; Deshpande *et al*., 2011). In this system, cells escape replicative senescence and proliferate indefinitely without telomeres, accumulating genomic deletions and amplifications, for example palindromic duplications (Maringele & Lydall, 2004b; Lee *et al*., 2008), hence the name. The factors facilitating their proliferation, despite of the extensive DNA damage, are still unknown.

Here we use the PAL system to identify mechanisms regulating the transition of cells lacking telomeres, to cells with extensive genomic modifications. We investigated nucleases (Exo1 and Mre11), checkpoint proteins (Rad24, Rad9 and Tel1), telomere‐associated proteins (Rif1, Rif2, Est2, Sir3, Yku70) and other factors (Ckb2) for a potential role. We report that Exo1 and Mre11 nucleases act synergistically with checkpoint proteins like Rad9 to inhibit escape from senescence. In survivors that managed to escape, Exo1 accelerates genomic rearrangements, whereas checkpoint proteins appear to lose the ability to detect telomere damage. In contrast, Rif1 facilitates the escape from cell cycle arrest of cells lacking telomeres or undergoing DNA double strand breaks (DSBs). The effect of Rif1 is consistent with an anti‐checkpoint mechanism. Our results are relevant for genomic modifications initiated by telomere‐free chromosome ends and by DSBs.

## Results

### Checkpoint pathways remain intact in cells proliferating without telomeres

**Figure 1:**
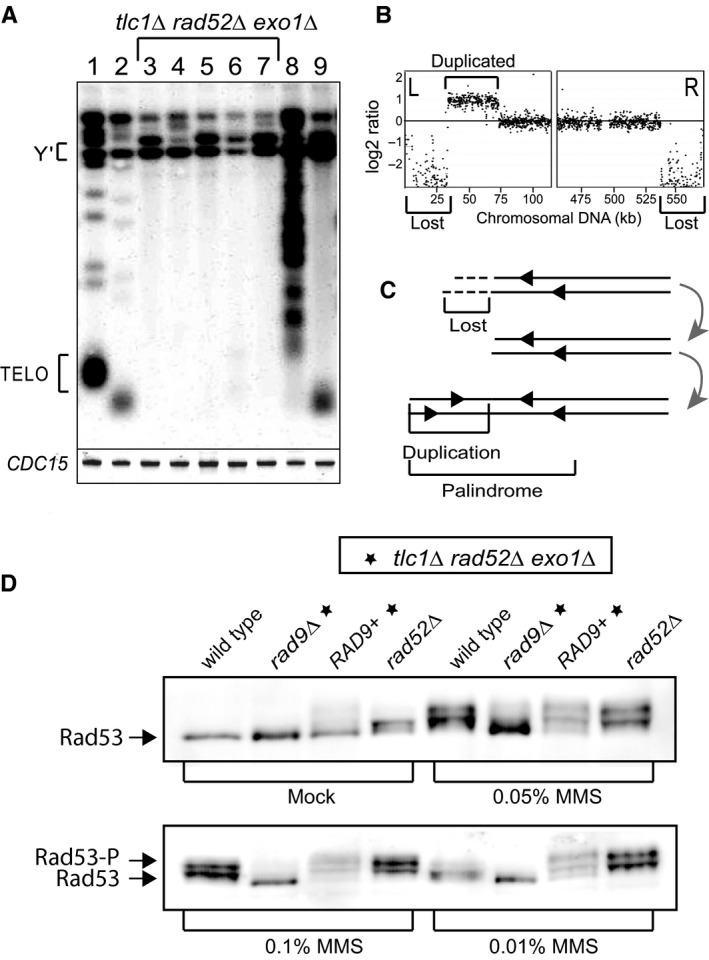
Cells proliferate without telomeres while checkpoint‐proficient. (A) Telomere blot showing restriction fragments corresponding to Y′ sub‐telomeres (Y′) and telomeres (TELO). Lane 1 shows the wild‐type. Lane 2, a *tel1∆* strain, mutation causing short, yet functional telomeres. Lanes 3–7 show five independent *tlc1∆ rad52∆ exo1∆* strains freshly escaped from senescence without telomeres. Lane 8 shows a *tlc1∆* strain with elongated telomeres; lane 9, a *tlc1∆ mre11∆* strain with amplified Y′ sub‐telomeres (type I survivor). The *CDC15* gene was detected as a loading control. (B) CGH analysis of chromosome V in a PAL survivor at passage 50. Each dot represents 100 nucleotides of nonrepetitive genomic DNA. Dots above the baseline indicate DNA amplification; below the baseline indicate DNA losses. (C) Diagram depicting the succession of events leading to the genomic modifications described in B. (D) Rad53 phosphorylation in cells exposed to different concentration of MMS for 4 h, or mock treated. Top left: mock treated; top right treated with 0.05% MMS. Bottom left: 0.1% MMS; bottom right: 0.01% MMS. Relevant genotypes are indicated above pictures, with additional gene mutations (e.g. the triple deletion‐mutation *tlc1∆ rad52∆ exo1∆*) indicated by stars.

PAL cells are yeast cells that contain neither of the two major mechanisms of maintaining telomeric DNA (telomerase or recombination). This is a good model for human somatic cells (lacking telomerase activity and rarely undergoing telomere recombination). Despite losing telomeres, PAL cells continue to proliferate for many generations with uncapped (free) chromosome ends (Maringele & Lydall, 2004b; Lee *et al*., 2008). A plausible hypothesis, explaining how cell division continues in the absence of telomeres, is that checkpoint pathways are inactivated. To test this hypothesis, we generated new PAL strains (PALs) and showed that they have lost most of the telomeric DNA (Fig. 1A). Fifty passages later, we performed comparative genome hybridization (CGH) and found that several kilobases of nonrepetitive DNA were lost from the chromosome ends, and that some of the end‐chromosomal regions appeared duplicated (Fig. 1B). The succession of these events in PALs was previously described (Maringele & Lydall, 2004b) and is summarized in a diagram (Fig. 1C), showing that PAL cells emerge from senescence and proliferate while losing chromosome ends. Occasionally, a duplication event takes place at a chromosome end, leading to the formation of a palindrome (e.g. a mirror image of an end‐terminal chromosome region, Fig. 1C). This event duplicates genes which otherwise will be lost, and it is so far the only mechanism known to keep PAL cells alive and proliferating.

To test the hypothesis that checkpoints must have been inactivated in PALs to allow for cell proliferation, we performed a standard yeast checkpoint activation assay, detecting the phosphorylated forms of the Rad53 checkpoint protein, in response to methylmetane sulphonate (MMS) and Phleomycin. We found that a range of MMS concentrations activated Rad53 in PAL cells to levels similar to those found in *rad52∆* and wild‐type cells (Fig. 1D). Phleomycin treatment gave similar results to MMS (data not shown). These indicate that PAL cells were checkpoint‐proficient. Interestingly, mock‐treated PAL cells also showed some Rad53 activation, which was rather modest, considering that they lacked telomeres. The Rad9 checkpoint protein was required for the Rad53 activation, since *rad9∆* PAL cells largely failed to activate Rad53, with or without MMS. We concluded that the Rad9–Rad53 checkpoint pathway remained intact in PAL cells. However, 32 telomere‐free chromosome ends (resembling to as many double strand breaks) did not massively activate this major checkpoint pathway. This result is remarkable because yeast cells usually activate the Rad9–Rad53 pathway in response to a single unrepaired DSB or to a lost telomere (Sandell & Zakian, 1993; Harrison & Haber, 2006) and raised the question of the mechanisms behind this checkpoint tolerance.

### Checkpoints and nucleases act differently to suppress PAL survivors

**Figure 2:**
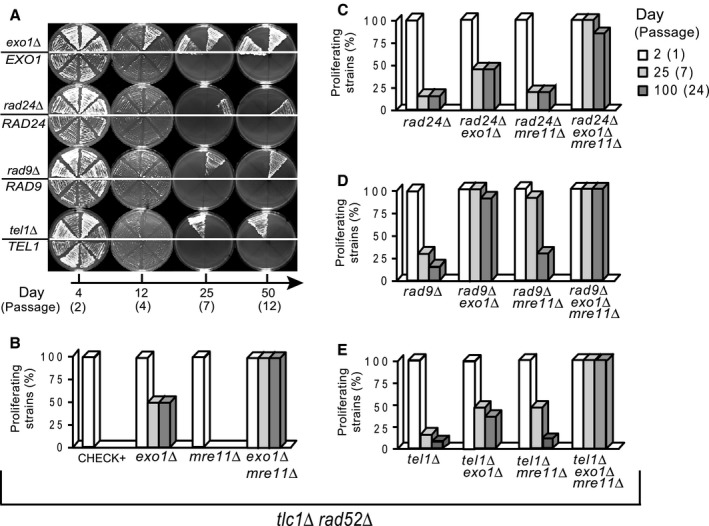
The effect of checkpoints and nucleases on escape from replicative senescence. At least 20 independent isogenic strains, taken directly from the germination plates, were propagated on a succession of fresh YPD plates, and photographed at the time points indicated below the pictures. (A) Representative plates, each with eight independent strains, photographed at 4, 12, 25 and 50 days. On the top half of each plate: either *exo1*∆ or checkpoint‐defective strains (*rad24*∆ *rad9*∆ and *tel1*∆.Other half: Exo1 and checkpoint‐proficient strains. All strains are *tlc1*∆ *rad52∆* (B–E) Columns represent the percentage of isogenic strains that escaped from senescence and were still proliferating at the time points indicated by day and passage number.

To address the mechanisms by which cells without telomeres, yet with intact checkpoint pathways continue to divide, we examined the effects of checkpoint and nuclease proteins on the ability of cells lacking telomeres to escape from senescence and proliferate long term. Numerous independent strains containing mutations affecting telomerase (*tlc1∆*) and recombination (*rad52∆*), in addition to other mutations in genes of interest, were serially propagated as in Figure 2(A). The fraction of isogenic strains proliferating at specific times is depicted in Figure 2(B–E). As previously reported (Maringele & Lydall, 2004b), an *exo1∆* mutation allowed 50% of *tlc1∆ rad52∆* strains to divide indefinitely, whereas an *mre11∆* mutation had no effect on its own, yet raised the fraction of proliferating *tlc1∆ rad52∆ exo1∆* strains to 100% (Fig. 2B).

We found interesting interactions between checkpoint, Exo1 and Mre11 proteins in opposing the emergence of cells without telomeres. Firstly, *EXO1+ tlc1∆ rad52∆* cells were able to generate PAL survivors, if they lacked any of the tested checkpoint proteins: Rad9, Rad24 or Tel1 (Fig. 2A). About 15–30% of *rad24∆*,* rad9∆* or *tel1∆* strains generated PAL survivors that proliferated for 100 days and longer (Fig. 2C–E). The *rad24∆* and *tel1∆* mutations appeared to be epistatic to *exo1∆* because the respective double mutants had similar fractions (50%) to *exo1∆* single mutants (Fig. 2C). In contrast, an *exo1∆* mutation drastically raised the proliferating fraction of *rad9∆* strains, from 30% to 100% (Fig. 2D). Similarly, an *mre11∆* mutation raised the proliferating fraction of *rad9∆* and *tel1∆* strains, however many of the resulting PALs perished by day 25 (Fig. 2C–E). Furthermore, an *exo1∆mre11∆* double mutation induced the highest proliferating fraction of 100%, irrespective whether strains were checkpoint‐proficient or defective (Fig. 2B–E).

In summary, checkpoint and nucleases interact to oppose the emergence of PAL survivors. Exo1 has the strongest, Tel1 the weakest effect. Mre11 has an effect only in the absence of Exo1 or checkpoint proteins. Rad24 seems to function in a pathway with Exo1, whereas Rad9 acts synergistically with either Exo1 or Mre11. Tel1 acts in a different pathway to Mre11, and possibly together with Exo1. These experiments show that checkpoint and nuclease proteins most often act in different pathways with synergistic effects to oppose the emergence of cells lacking telomeres.

### Exo1 causes extensive gene deletion and poor growth phenotype in PALs

**Figure 3:**
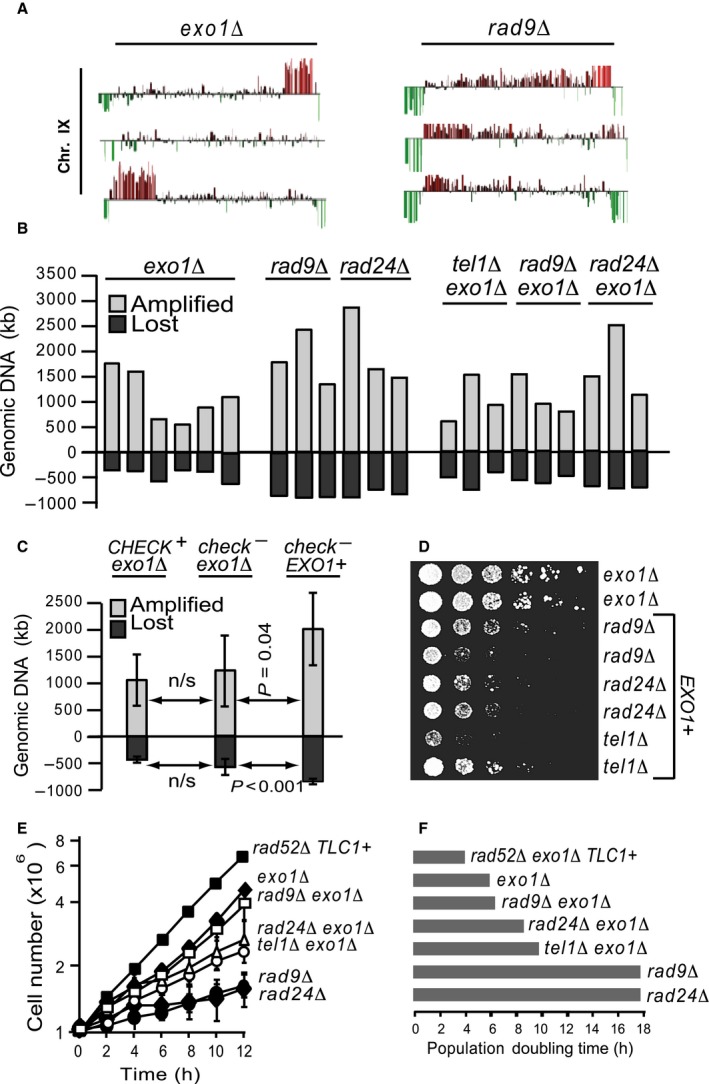
Exo1 accelerates genomic alterations and inhibits PAL proliferation. All strains are *tlc1∆ rad52∆*, unless otherwise stated. (A) Examples of chromosomal alterations (detected by CGH) affecting chromosome IX in three independent *exo1∆* PALs (left cluster) and *rad9∆* PALs (right cluster). Spikes above the baseline indicate DNA amplification; below the baseline indicate DNA losses. (B) Cumulative genomic alterations in 200 days PALs. Light columns (above the baseline) represent the amount of amplified DNA *per* strain (in kb). Dark columns (below the baseline), the amount of deleted DNA. Relevant mutations are indicated above the columns. (C) The average amount of DNA amplified and deleted in PALs presented in B, clustered according to checkpoint‐proficiency in *CHECK* ^*+*^ (e.g. checkpoint‐proficient) and *check* ^−^ (e.g. checkpoint‐defective *rad9∆* and *rad24∆*). Error bars are the standard deviation. (D) Droplets of five‐fold serial diluted cultures of 50 days PALs were spotted onto plates and incubated for 5 days at 25 °C. Relevant mutations are indicated. (E) Growth of different PALs and telomerase‐positive controls (*rad52∆ TLC1+*), diluted to 1 × 10^6^ cells ml^−1^ at time 0 and incubated for 12 h at 25 °C. Error bars are the standard deviation. (F) Each horizontal bar indicates the average population doubling time, e.g. the amount of time (h) required for cultures analysed in E to double in cell number.

Our data suggested that Exo1 acts in a pathway with Rad24. However, Exo1 must also act independently of Rad24, since it has a stronger effect than Rad24, in eliminating cells lacking telomeres. To determine the Rad24‐independent roles of Exo1, we examined the genome of numerous PAL survivors, using CGH. We found that different genetic backgrounds had quantitatively different rearrangements. Examples of our CGH analyses show losses or duplications of gene loci towards chromosome ends, in three independent *exo1∆* and *rad9∆* PAL strains (Fig. 3A). All our data are summarized in Figure 3(B). By passage 50, *exo1*∆ PALs had lost an average of 430 kb DNA from chromosome ends (Fig. 3B,C). In contrast, *EXO1+ rad9*∆ and *rad24*∆ PALs had lost twice as much chromosomal DNA, in average 850 kb. This comparison shows that accelerated loss of DNA was Exo1‐dependent.

Loss of genetic material will cause cell death if essential genes are lost, and therefore increases the selective pressure for other genomic changes, such as duplications (palindromes). Consistent with this, we found that *EXO1+* PALs amplified significantly more genomic DNA than *exo1*∆ PALs (Fig. 3B), on average 1950 kb vs. 1050 kb (Fig. 3C). Moreover, the accelerated genomic losses and amplifications were largely checkpoint‐independent, since checkpoint‐defective and checkpoint‐proficient *exo1*∆ PALs showed similar quantitative alterations (Fig. 3B,C). Therefore, Exo1 contributes to the rapid loss of genes and the subsequent gene amplification induced by the absence of telomeres, whereas checkpoint pathways do not have such an effect.

The Exo1‐induced genomic instability led to a decrease in fitness of PALs, since *EXO1+* (checkpoint‐defective) PALs had a poor growth phenotype on plates (Fig. 3D) and in liquid culture (Fig. 3E). Their population doubling time was 18 h, compared to only 6 h for *exo1∆* checkpoint‐proficient and to 7–8 h for *exo1∆* checkpoint‐defective PALs (Fig. 3E,F). Interestingly, checkpoint‐proficient *exo1∆* PALs also grew slightly better than *exo1∆* checkpoint‐defective PALs (Fig. 3E,F). This suggests that checkpoint pathways confer a growth advantage to PAL cells, most likely by facilitating repair of the intrinsic damage. In conclusion, in cells proliferating without telomeres, Exo1 increases the gene deletion and duplication and decreases the fitness. In contrast, the checkpoint pathways do not affect the end‐chromosomal gene deletion and duplication, but instead confer a subtle growth advantage to these challenged cells.

### Restitution of checkpoint or nuclease activities eliminates PAL survivors

A plausible hypothesis, explaining how cells lacking telomeres proliferate well, is that they acquire some unknown, growth‐facilitating mutations. If this were the case, then perhaps nucleases and checkpoints would become less relevant for opposing proliferation of PAL survivors. To test this hypothesis, we transformed long‐time proliferating PALs with a centromeric vector, containing one copy of *EXO1*,* MRE11*,* RAD9*,* RAD24* or *CHK1* respectively, under control of their own promoters. *CHK1* encodes a downstream checkpoint kinase. We could test only *exo1∆* PALs in this way because the less fit *EXO1+* PALs did not survive transformation.

**Figure 4:**
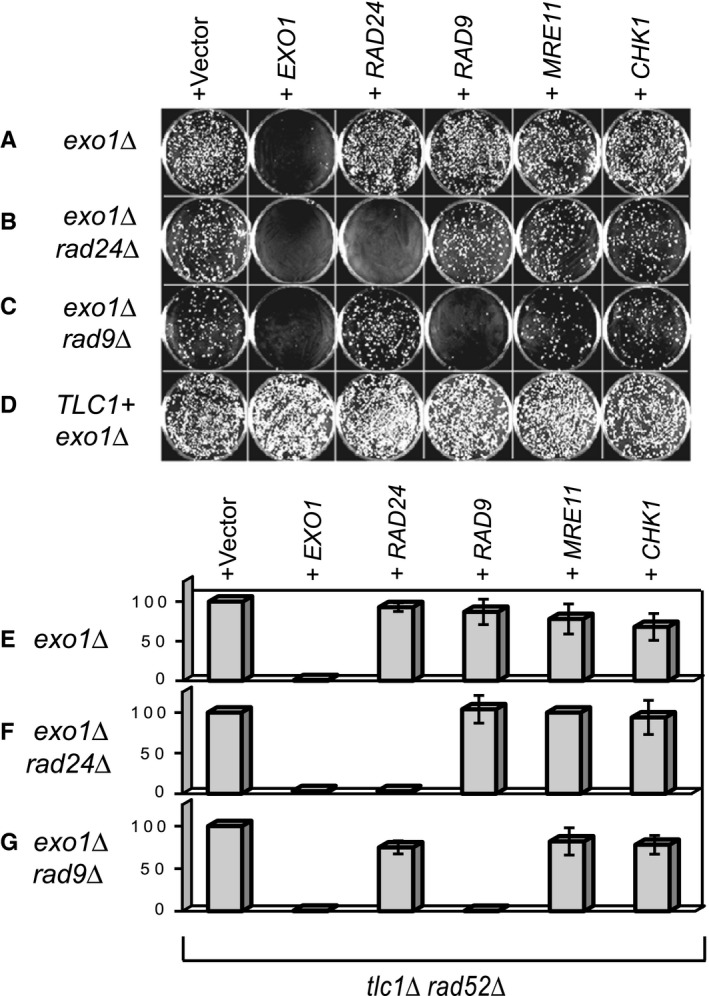
Cell proliferation remains dependent upon mutation(s) that permitted escape from senescence. 200 days PALs (*tlc1∆ rad5□∆*) with additional mutations (indicated on the left) and telomerase‐positive controls were transformed with DNA vectors caring genes of interest (indicated above). (A–D) Representative plates incubated for 7 days following the transformation. (E–G) Columns represent the percentage of colonies obtained after transformation with genes indicated above plates/columns, relative to the percentage of colonies obtained after transformation with the empty vector. Error bars are the standard deviation between measurements in six independent PALs.

We found that transformation with *EXO1* eliminated the vast majority of *exo1∆* PAL cells, irrespective of their checkpoint status (Fig. 4A–C,E,F), suggesting that Exo1 caused loss of essential genes. Moreover, *RAD24* transformed into *exo1∆rad24∆* PALs and *RAD9* into *exo1∆rad9∆* PALs also eliminated growth (Fig. 4B,C,F,G), suggesting that checkpoint‐proficiency was restored, leading to cell cycle arrest. Thus, PAL survivors that have evolved in the absence of Rad9 or Rad24 rely on the continuous absence of these checkpoint proteins. Reintroduction of *RAD24*,* RAD9*,* MRE11* or *CHK1* did not affect proliferation of *exo1∆* PALs that were not originally mutated in the corresponding gene (Fig. 4). We infer that mutations inactivating checkpoints are not frequent in PAL survivors. In conclusion, proliferation of PAL cells lacking telomeres remains dependent upon the continued inactivation of nucleases and/or checkpoint pathways that allowed them to arise.

### Rif1 acts differently to Rif2 and Mre11 during replicative senescence

**Figure 5:**
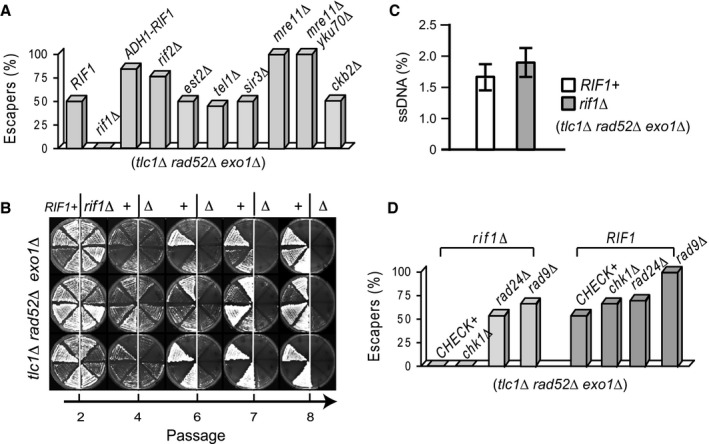
Rif1 is required for proliferation of cells losing telomeres. All strains are *tlc1∆ rad52∆ exo1∆*, unless otherwise stated. (A) Columns represent the fraction of PALs escaping replicative senescence and proliferating at passage 15. Relevant mutations are indicated above columns. The *yku70∆ mre11∆* is *TLC1+*, yet senesce and escape as described (Maringele & Lydall, 2004b). (B) Several newly germinated *rif1∆* strains (on the right half of each plate) and the same number of *RIF1+* controls (left half), were propagated every 5 days on a succession of plates and photographed at the indicated passage. (C) Single‐stranded DNA measured by QAOS in senescent (passage 4) *RIF1+* and *rif1∆* strains. Error bars are the standard deviation between three measurements performed in sub‐telomeric regions. (D) As in A, except that PALs in the left cluster are *rif1∆*; in the right cluster, they are *RIF1+*. Additional checkpoint mutations are indicated above each column.

We have shown that checkpoint pathways remain intact in PAL survivors. However, they fail to detect cells dividing with telomere‐free chromosome ends. A plausible hypothesis is that some of the proteins usually associated with telomere sequences could become associated with telomere‐free chromosome ends, in a DNA sequence‐independent manner, thus creating ‘epigenetic telomeres’. To test this hypothesis, we screened a number of nonessential telomere‐associated proteins for their input in PAL survivor formation (*tlc1∆ rad52∆ exo1∆* strains) We tested Tel1, Rif1, Rif2, Est2, Sir3, Yku70 and Mre11 (Fig. 5). We also tested the casein kinase component Ckb2 because it is involved in checkpoint adaptation, a process by which checkpoint pathways adapt to and ‘ignore’ an unrepaired DNA double strand break (Toczyski *et al*., 1997; Pellicioli *et al*., 2001).

Interestingly, Rif1 was the only tested gene that was important for generation of PAL survivors, since a *rif1*∆ mutation completely abolished the escape (Fig. 5A). Conversely, overexpression of *RIF1* using the *ADH1* promoter increased the emergence of PAL survivors, from 50 to about 80% (Fig. 5A). In contrast, inactivation of Est2, Tel1, Sir3, Ckb2 or Yku70 did not affect the ability of *exo1∆* strains to escape senescence. Interestingly, inactivation of Rif2 increased the escape fraction, from 50% to 75%. In this respect, the Rif2 was similar to Mre11, and opposite to Rif1 (Fig. 5A). Previously, it was found that Rif2 and Rif1 have synergistic effects in inhibiting telomerase (Wotton & Shore, 1997; Levy & Blackburn, 2004) and yet opposing effects in regulating the proliferation of telomere‐dysfunctional *cdc13‐1* cells (Addinall *et al*., 2008; Xue *et al*., 2011). In conclusion, Rif1 is unique among many telomere‐associated proteins in facilitating proliferation of cells lacking telomeres, whereas Rif2 and Mre11 have the opposite effect to Rif1.

### Rif1 is essential for proliferation of cells with telomere losses

To confirm the requirement for Rif1 in PAL survivors, we inoculated several plates, each with four *RIF1*+ PALs (the right semicircle) and four *rif1∆* PALs. Representative plates are shown in Figure 5(B). All strains had an escape‐facilitating *exo1∆* mutation. To our surprise, not a single colony out of 80 independent strains emerged from senescence in the absence of Rif1. These data confirms that Rif1 is absolutely required for the emergence of PAL survivors. Rif1 might have facilitated escape from senescence by inhibiting the checkpoint responses, the chromosome end resection, or the chromosome end fusions. Inhibition of resection played only a minor role, since senescent *tlc1∆ rad52∆ exo1∆* strains accumulated similar levels of sub‐telomeric single‐stranded DNA, irrespective of whether they were *RIF1+* or *rif1*∆ (Fig. 5C). Moreover, deletion of *LIG4*, encoding a ligase essential for covalent fusions, did not facilitate *rif1∆* cells to escape from senescence, suggesting that chromosome fusion was not responsible for their inability to form PAL survivors (data not shown).

If the critical role of Rif1 in telomere‐free cells was due to inhibiting the checkpoint responses (e.g. an ‘anti‐checkpoint’ function), then inactivating relevant checkpoints should alleviate the importance of Rif1. To test this hypothesis, we analysed the genetic interaction between checkpoint genes and *RIF1*. Importantly, we found that checkpoint inactivation (e.g. *rad9∆* or *rad24∆* mutations) enabled *rif1∆ exo1∆*PALs to proliferate long term (Fig. 5D). In contrast, a *chk1∆* mutation had no apparent effect (Fig. 5D). The difference between checkpoint effects was most likely due to the essential role of Rad9 and Rad24 in maintaining replicative senescence (Deshpande *et al*., 2011), whereas Chk1 may have a limited role. In conclusion, the critical role for Rif1 in cells lacking telomeres manifests when relevant checkpoint pathways are intact. Therefore, our data suggest that the Rif1 activity is consistent with an anti‐checkpoint effect in cells proliferating without telomeres.

### Rif1 associated with DSB facilitates the checkpoint adaptation

**Figure 6:**
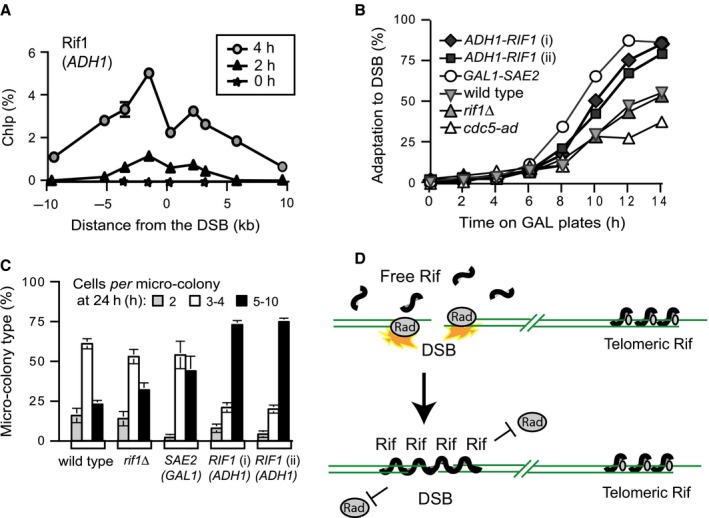
Rif1 protects telomere‐free chromosome ends. (A) Association of Rif1‐HA, expressed from the *ADH1* promoter, with the margins of a DSB in strains with a JKM139 background, expressing the HO‐nuclease from a galactose‐induced promoter (Lee *et al*., 1998). Strains were grown overnight on raffinose; galactose was added at time 0 and samples collected every second hour. Galactose induces GAL‐HO‐nuclease to cut at the MATa locus. Because the donor locus is missing, repair by recombination is prevented (Lee *et al*., 1998). The numbers on the *X*‐axis indicate the distance from the DSB. The legend indicates the time (h) in galactose. (B) The fraction of cells that adapted, e.g. escaped from arrest by producing at least another large bud/cell. JKM139 and derivates from overnight raffinose cultures were incubated on galactose at 30 °C, to induce a DSB. *ADH1‐RIF1* (i) and (ii) are independent strains with *RIF1* under the *ADH1* promoter. After 4 h, large‐budded cells were separated by sonication, transferred to galactose plates and incubated at 30 °C. Cells were examined every second hour by microscopy. (C) As in B, except that the fraction of micro‐colonies formed after 24 h on galactose plates is presented. The micro‐colonies consist of the following number of cells and buds: 2 (grey columns), 3–4 (white) and 5–10 (black). (D) Cartoon explaining the effect of Rif1 in cells with a chromosome break.

Rif1 was shown to associate with single‐stranded DNA regions in *cdc13‐1* strains, where it inhibited RPA and checkpoint proteins (Xue *et al*., 2011). Therefore, it is plausible that Rif1 associates with chromosome ends lacking telomeres in PAL survivors, thus inhibiting their detection by checkpoint sensors and facilitating proliferation. This hypothesis is difficult to test in PAL survivors directly, due to the on‐going erosion of chromosome ends. However, we tested this hypothesis at an HO‐induced double strand break (DSB) at the MAT locus in JKM139‐derived cells unable to repair the break by homologous recombination, as described previously (Lee *et al*., 1998). Normally yeast Rif1 fails to significantly associate with a DSB (Xue *et al*., 2011), whereas mammalian Rif1 co‐localizes with DSBs (Buonomo *et al*., 2009). However, when we increased the amount of Rif1 in cells, by expressing *RIF1‐HA* from the stronger *ADH1* promoter, we could detect Rif1 binding near the DSB (Fig. 6A).

We next tested whether Rif1 affected cell proliferation. Almost all cells arrested proliferation within 4 h following induction of the DSB, but later underwent checkpoint adaptation (e.g. they started to divide again), consistent with previous reports (Lee *et al*., 1998; Pellicioli *et al*., 2001). Up to 50% of the GAL‐HO cells (marked as wild‐type) adapted by 14 h after the DSB induction (Fig. 6B), whereas only 25% *cdc5‐ad* cells, known as adaptation‐defective (Toczyski *et al*., 1997) adapted. In contrast, 80% *ADH1‐RIF1* cells adapted by 14 h (Fig. 6B), similarly to the *GAL1‐SAE2* cells, which are known to adapt very efficiently (Clerici *et al*., 2006). Moreover, *ADH1‐RIF1* cells formed larger micro‐colonies on agar plates. About 75% *ADH1‐RIF1* cells generated colonies of 5–10 cells 24 h after the DSB induction, compared to only about 25% wild‐type cells (Fig. 6C). These data indicate that in the presence of a DSB, cells overexpressing Rif1 divide more than wild‐type cells and that Rif1 can bind DSBs and act in an anti‐checkpoint (or pro‐adaptation) manner. Since telomere‐free chromosome ends in PAL survivors are similar to double strand breaks, we propose that Rif1 forms ‘epigenetic telomeres’ that inhibit the checkpoint responses and thus drives the escape from replicative senescence.

## Discussion

The factors permitting cells with short or absent telomeres to proliferate are little understood. Using yeast cells, we show that complex genetic interactions between DNA damage responses factors determine the efficiency by which cells emerge from senescence without telomeres. We show that the nuclease Exo1, whose activity was considered incompatible with PAL survival (Maringele & Lydall, 2004b), actually inhibits survival through checkpoint‐dependent and checkpoint‐independent roles. The DNA damage checkpoint proteins Rad9, Rad24 and Tel1 are also inhibiting the PAL survivor emergence, but to a lesser extent than *EXO1*. An *exo1∆* mutation in combination with either *mre11∆* or *rad9∆* provides the most efficient route to PAL survivor formation, indicating that Rad9, Exo1 and Mre1 act in different pathway with synergistic effect to inhibit the emergence of PALs. In contrast, Rad24 and Tel1 seem to function in the same pathway as Exo1. These data support the ‘Vicious Cycle’ model of replicative senescence, which stipulates that the continuously alternating activities of at least two pathways, involving Exo1‐Rad24 and Rad9‐Polymerase epsilon respectively, are required to maintain replicative senescence (Deshpande *et al*., 2011).

**Figure 7:**
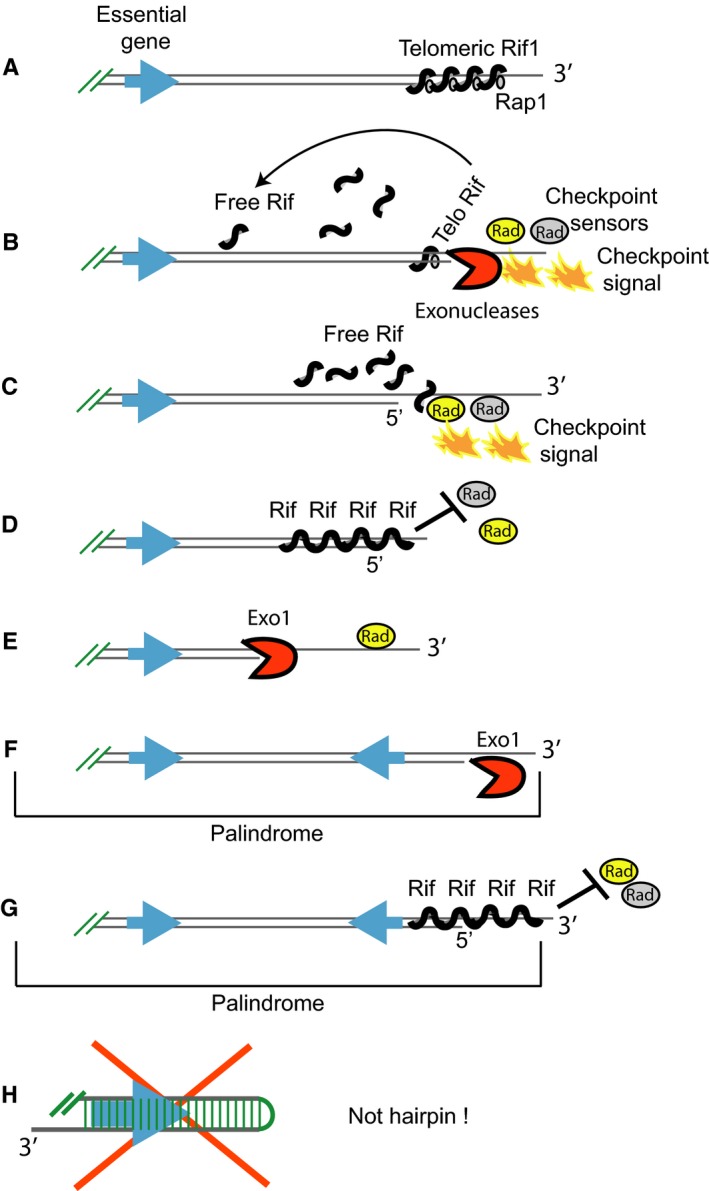
The role of Rif1 and Exo1 at chromosome ends in cells proliferating without telomeres. (A) Cartoon depicting Rif1 associated with (sub)telomeric sequences through Rap1, at a functional chromosome end. (B) A short telomere has lost its capping function. Checkpoint sensor proteins and exonucleases detect and process the chromosome end, generating single‐stranded DNA (ssDNA) and ‘checkpoint signals’ leading to downstream checkpoint responses and senescence. Rap1 and Rif1 are released from single‐stranded (sub)telomeres. (C) ‘Free’ Rif1 associates with chromosome ends, replacing and/or displacing checkpoint sensors. (D) Arrays of Rif1 proteins form anti‐checkpoint ‘shields’ at chromosome ends. Cells may escape senescence, if Exo1 is inactive. Inactivation of Exo1 keeps ssDNA levels low, meaning that less Rif1 proteins are required for shielding ssDNA from checkpoint sensors. The checkpoint signal is therefore effectively extinguished. (E) Chromosome ends in cells with active Exo1 suffer from extensive degradation. Such cells can still escape from senescence when checkpoints are disabled. We suggest that a Rif1 shield may not form in checkpoint‐defective PAL cells because such cells re‐enter senescence when their checkpoint‐proficiency is restored (Fig. 4). (F) The structure of a palindromic chromosome end in *EXO1+* checkpoint‐defective PALs. The Exo1‐driven chromosomal degradation reaches more rapidly essential genes and kills the cell. Cells that duplicate the endangered essential genes by forming palindromes survive and proliferate. Consequently, there are many more palindromes in *EXO1+* PALs than in *exo1∆* PALs. (G) The structure of a palindromic chromosome end in *exo1∆* checkpoint‐proficient PALs. We suggest that palindromic ends consist of DNA with a 3′ overhang, protected against checkpoints by Rif1 shields. (H) Do palindromes form hairpins? If they did, hairpins would appear as nonduplicated DNA by CGH. This is because a hairpin is a palindrome that has lost one of its DNA strands, whereas the complementary halves of the other DNA strand annealed to each other. Therefore, we are certain that palindromes detected as DNA duplications are not hairpins. However, a small fraction of palindromes may form hairpins. This is because weak, nonduplicated DNA bands (‘half‐sized’ bands) were detected by Southern blotting, in addition to strong palindrome bands, in clones of PALs (Maringele & Lydall, 2004b). The ‘half‐sized’ bands were suggested to emerge when palindromes formed cruciform structures and these were cut in half by resolvase. However, it is also possible that the ‘half‐sized’ bands are hairpins.

To determine the checkpoint‐independent role of Exo1 in cells escaping senescence, we examined the karyotype of numerous independent PAL survivors. We observed an Exo1‐dependent loss of genes, as well as gene duplication events, in cells lacking telomeres. Exo1 has been previously documented to facilitate or inhibit chromosomal duplication following other type of insults, through a checkpoint‐dependent (Kaochar *et al*., 2010) or homology‐directed repair (HDR) dependent mechanism (Štafa *et al*., 2014). However, the palindrome formation in PAL cells is both HDR‐independent (PALs are *rad52∆*) and checkpoint‐independent (in *rad9∆* and *rad24∆* strains) and therefore occurs by a different mechanism. We propose that the Exo1‐driven chromosome degradation increases the selective pressure to form palindromes at chromosome ends (Fig. 7E,F). We suggest that mammalian Exo1 or other nucleases could play a role during the malignant transformation of DNA damaged cells. For example, inactivation of Exo1 could facilitate escape from senescence and proliferation of mammalian cells with telomere defects. However, deletion of Exo1 did not appear to be a risk factor for cancer in mice lacking telomerase (Schaetzlein *et al*., 2007). Conversely, Exo1 may instead facilitate genomic alterations relevant to carcinogenesis in DNA‐damaged human cells, similarly to its role in PAL cells. In support of this, a potential link between Exo1 polymorphisms and premalignant lesions (colorectal adenoma) in tobacco smokers has been reported (Gao *et al*., 2011).

PAL survivors are able to proliferate with ‘free’ chromosome ends, e.g. with extensive DNA damage. This strongly suggests that they must have lost their checkpoint control. Amazingly, this was not the case because PAL cells had a robust DNA damage response after treatment with the alkylating agent MMS. Moreover, we found no evidence that *RAD9, RAD24, MRE11* or *CHK1* checkpoint genes were inactivated. In fact, checkpoint‐proficient PALs grew slightly better than checkpoint‐defective homologues, presumably because checkpoints pathways are helping preserving the viability of cells. We conclude that checkpoint pathways are very important during senescence, when they cooperate and synergize with nucleases, however, once survivors emerge, they become tolerant to telomere losses and fail to inhibit proliferation.

To understand the mechanism allowing cells lacking telomeres to proliferate, despite intact checkpoint pathways, we screened several telomere‐relevant genes, for a potential role in this process*. RIF1*, but not *RIF2*,* EST2*,* SIR3*,* YKU70*,* CKB2* or *MRE11*, was the gene that helped PAL survivors grow. We found that in the absence of Rif1, cells cannot escape the replicative senescence barrier. Moreover, when Rif1 was overexpressed, it facilitated escape from senescence, and also the proliferation of cells with an internal DSB, with which Rif1 associated. These data indicate that Rif1 is important for PAL cells, most likely because it has the potential to protect DNA ends from the DNA damage responses. We propose that Rif1 displaces checkpoint sensors at the unrepairable DSB, similarly to its effect in *cdc13‐1* uncapped cells (Xue *et al*., 2011). In consequence, cells escape arrest and proliferate for longer (Fig. 6D). Increased levels of Rif1 appear to be important for this effect. Consistent with this, the chromosome arm containing Rif1 appears duplicated in 2/3 of PALs analysed by CGH (data not shown).

To explain the roles of Rif1 and Exo1 in cells that escaped from senescence without telomeres and formed palindromes, we propose the model presented in Figure 7. During replicative senescence, the amount of ‘free’ Rif1 increases with telomere losses because it is released from its association with Rap1 and telomeres (Fig. 7B). Rif1 may associate with telomere‐free chromosome ends, acting as an anti‐checkpoint shield and thus allowing cells to escape from senescence (Fig. 7C,D). By permitting cells with DNA damage to divide, Rif1 becomes responsible for the genomic instability and chromosomal alterations affecting these cells. Rif1 shields are also expected to protect the end of palindromes formed in PALs (Fig. 7G). However, Rif1 shields are not required and may not actually form at chromosome ends of checkpoint‐defective PAL cells (Fig. 7E,F) since such cells stop dividing (re‐enter senescence) when checkpoint‐proficiency is restored (Fig. 4).

Interestingly, high levels of Rif1 in embryonic stem cells lead to genomic instability and malignant transformation (Li *et al*., 2015). Similarly, elevated levels of Rif1 were found in breast cancer and teratocarcinomas (Wang *et al*., 2009; Li *et al*., 2015). Since mammalian Rif1 participates in suppressing the HDR repair pathway in the G1 phase (Chapman *et al*., 2013; Di Virgilio *et al*., 2013; Escribano‐Díaz *et al*., 2013; Zimmermann *et al*., 2013), it was suggested that too much Rif1 drives illicit and error‐prone DSB repair, which alters the genome. However, our study shows that Rif1 can drive genomic instability in the absence of DSB repair. This is because Rif1 facilitated the proliferation of cells with an unrepairable broken chromosome, and that of senescent cells lacking HDR. Whereas cells have evolved mechanisms that ensure that little or no DNA damage is passed onto their progenies, Rif1 could be the key factor used by genomically compromised cells, for example senescent cells, to bypass such mechanisms and resume proliferation.

## Experimental procedures

### Yeast strains and proliferation assays

All strains are derivates of W303 *RAD5* ^*+*^. The *tlcl∆rad52∆* strains other relevant mutations originate from the DLY2150 diploid, heterozygous for the following genes: *TLC1/tlc1∆::HIS3*,* RAD52/rad52∆::TRP1*,* EXO1/exo∆::LEU2* and *MRE11/mre11∆::URA*. We additionally deleted *TEL1*,* RAD9* or *RAD24* in W303, by converting them into G418‐MX cassettes. To confirm heterozygosity for the *TEL1/RAD9/RAD24* genes, colonies from G418 transformation plates were analysed by PCR. The following diploid strains were obtained: DLY2693 (heterozygous for *TEL1*), DLY2697 (heterozygous for *RAD9*) and DLY2698 (heterozygous for *RAD24*). Diploid cells were sporulated and haploids selected by random spore analysis. Then, 20 isogenic haploids were individually tested by PCR to re‐confirm deletion of *TEL1/RAD9/RAD24* genes and propagated at 25 °C. Cells were grown in YPD medium supplemented with adenine at 50 mg l^−1^, unless otherwise specified. For replicative senescence assays (Figs 2A and 5B), cells taken directly from germination plates were propagated every second day until they became senescent, by pooling circa 1 × 10^7^ cells with a toothpick and streaking them onto fresh YPD plates, as previous (Maringele & Lydall, 2004b). Strains escaping from senescence (PAL survivors) were propagated every 4–5 days. Serial dilutions (Fig. 3) were performed as previously described (Maringele & Lydall, 2002).

### Immunoblotting

Cells were diluted to 1 × 10^7^ cells ml^−1^ and treated with different concentration of MMS for 4 h or mock treated. Protein extracts were prepared by a trichloroacetic acid (TCA) method and separated on SDS‐PAGE and transferred to PVDF membranes. Membranes were incubated with polyclonal anti‐Rad53 (ab104232 Abcam, Cambridge, UK). Southern blottings detecting telomeres, sub‐telomeres and *CDC15* gene were performed as described (Maringele & Lydall, 2004a). Shortly, DNA digested with Xho1 was separated on a gel, transferred to a membrane, UV‐cross‐linked and hybridized with a TG probe or with a *CDC15* probe. Hybridization was detected using a nonradioactive detection kit (Roche, Switzerland).

### Single‐stranded DNA

Single‐stranded DNA measurements were performed by QAOS and analysed by quantitative PCR in Y’ sub‐telomeres as previously described (Maringele & Lydall, 2002).

### Comparative genome hybridization

Micro‐array probes (40–70‐mer oligo‐nucleotides) representing 6250 ORFs in the *S*. *cerevisiae* genome (MWG) were printed onto Aldehyde+ slides (Genetix, New Milton, UK). Sample and reference DNA were random labelled using a BioPrime^®^ Array CGH Genomic Labelling Module (Invitrogen, MA, USA) and Cy5 or Cy3 conjugated dUTP (Amersham). The efficiency of each labelling reaction was quantified using Nanodrop ND‐1000, then 50 pmol of labelled target material was competitively hybridized to arrays for at least 18 h at 62 °C using M‐Series Lifterslips (Erie Scientific, Portsmouth, UK). Following washes, arrays were immediately scanned and analysed using Genepix 6 and a 4000B reader (Axon Instruments, CA, USA). Spots of irregular shape, containing high background or hybridization artefacts were flagged and omitted from further analysis. Data were then normalized using ratio‐based normalization, so that the mean of the ratio of medians was equal to one. Data were then exported into Aquity 4.0 for further analysis. Unlogged medians of PAL survivor/wild‐type ratio of values were used to draw chromosome plots in Acuity 4.0 using ‘Caryoscope’ mode. ORFs with a ratio between 0.01 and 0.5 were considered deleted, whereas ORFs with a ratio between 1.5 and 2.5 were considered duplicated. To avoid artefacts, we considered a chromosomal region to be amplified when at least three adjacent ORFs had ratio values of at least 1.5. Several CGH analyses, including the one presented in Figure 1(B), were performed by Roche Nimblegen using a 385K whole *S. cerevisiae* genome‐tiling array (385 000 probes, catalog number B2436001‐00‐01 2007‐05‐08 SCER WG CGH).

### Yeast transformation

For plasmid transformation, each strain was grown in liquid YPD, cells collected and divided into equal samples, each to be transformed with a different plasmid. Plasmids were centromeric, derived from pRS416, with one copy of the following genes under their own promoters (e.g. 500–700 bp of sequence upstream of the open reading frames): *EXO1* (pDL1034), *MRE11* (pDL1041), *RAD9* (pDL847), *RAD24* (pDL749) and *CHK1* (pDL928). Vector PRS416 (pDL13) was the negative control. The functionality of each exogenous gene was demonstrated in strains with uncapped telomeres (data not shown). Transformed strains were plated onto selective plates and incubated at 25 °C. Plates were photographed after 7‐day incubation.

### Chromatin immunoprecipitation

Chromatin immunoprecipitation (ChIP) was carried out as previously described (Xue *et al*., 2011). The association of Rif1‐HA with chromatin around a DSB was detected with rat monoclonal anti‐HA (11867423001; Roche). Cell extracts were also treated with anti‐goat antibodies (sc‐2033; Santa Cruz, CA, USA) to assess the background cross‐linking. For each time point, the background normalized to the input was subtracted from the immunoprecipitated DNA, also normalized to the input. Input, immunoprecipitated DNA and background were quantified by real‐time PCR (StepOne Plus; Applied Biosystems, CA, USA) using genomic DNA standards.

## Author contributions

YX and IGI performed some of the experiments. MEM performed the CGH and analysed the data. LM conceived the experiments, performed some of them, supervised YX and IGI and wrote the manuscript. DL supervised LM initially.

## Funding

No funding information provided.

## Conflict of interest

This work was supported by a Wellcome Trust Career Development Fellowship (award no. 81164).
